# Long-term compliance of vaginal pessaries: Does stress urinary incontinence matter?

**DOI:** 10.1097/MD.0000000000015063

**Published:** 2019-04-05

**Authors:** Ming-Fang Hsieh, Hsiao-Wen Tsai, Wen-Shiung Liou, Ching-Chuan Lo, Zi-Han Lin, Ya-Fen An, Hsin-Yin Lin

**Affiliations:** a Department of Obstetrics and Gynecology, Kaohsiung Veterans General Hospital, Kaohsiung; b School of Medicine, National Yang-Ming University, Taipei; c Department of Nursing, Shu-Zen Junior College of Medicine and Management, Kaohsiung, Taiwan.

**Keywords:** compliance, pelvic organ prolapse, stress urinary incontinence, vaginal pessary

## Abstract

Vaginal pessary treatment for pelvic organ prolapse (POP) is relatively safe and cost-effective. Since long-term use is an important key to keep the benefit of pessary treatment, we would like to investigate the factors which might affect the compliance of vaginal pessaries. In this retrospective study, 65 women were included, and we found poor compliance in women with severe stress urinary incontinence (SUI) after reduction (1-hour pad test >10 gm vs ≦10 gm, 57.1% vs. 84.3%, *P* = .027). Besides, women younger than 60 years-old also had poor compliance (age ≦60-year-old vs >60-year-old, 58.3% vs 83.0%, *P* = .04). Other factors such as POP stage, history of hysterectomy, and types of pessaries, did not show significant influence on the long-term compliance in this study. Therefore, to evaluate the severity of SUI after reduction before providing pessary treatment is important to predict long-term compliance. Meanwhile, long-term pessary treatment seems to be more acceptable to elderly patients.

## 1 Introduction

Pelvic organ prolapse (POP) affects millions of women's quality of life and the prevalence is expected to increase due to the aging of the population.^[1]^ Women with POP usually complain of vaginal bulging and various urinary and bowel symptoms, such as stress urinary incontinence (SUI), constipation, and sexual dysfunction.^[2,3]^ Surgical treatment such as sacrocolpopexy remains the standard of care, and transvaginal mesh surgery may be performed in selected patients. However, there are still a large proportion of patients who are medically contraindicated for surgery or not willing to receive surgery for POP.^[4]^ In addition, the chance of repetitive surgery due to recurrence of POP or complications is high, and the reoperation rate using mesh kits was estimated to be up to 8.5%.^[5]^ Therefore, nonsurgical intervention such as vaginal pessary treatment should always be considered first since it is a safe and effective alternative to manage POP.^[6,7]^

For women with symptomatic POP, Abdool et al reported similar improvement when they were treated with vaginal pessaries or surgery for 1 year.^[7]^ It was also reported after 1 year of pessary treatment, the progression of POP could be prevented.^[8]^ Thus, the treatment success relies on patients’ adherence to the vaginal pessaries. The 3 months short-term compliance of vaginal pessary treatment is ranging from 41% to 76%^[9–11]^ and the reasons of failure varied, including young age, previous hysterectomy, SUI, and the severity of POP.^[12–15]^ However, there is little data available regarding the factors that influence the long-term compliance. Since consistency is important, it is worth to investigate the reasons why patients quit vaginal pessaries.

According to the Women's Health Initiative studies, urinary symptoms are the most common and bothersome symptoms to women with POP.^[14,16]^ Pessary treatment may ease urinary frequency, urgency, and symptoms of bladder outlet obstruction of the patients, and thus, these patients may have good compliance. But to those who have symptoms of SUI, their urinary leakage may become worse after reduction of POP with pessaries. As a result, they may give up pessary treatment and seek for surgical intervention. Therefore, we hypothesized that severe SUI after reduction may result in poor long-term compliance.

The aim of our study was to determine if severe SUI after reduction or any other factors could affect long-term compliance of vaginal pessaries for women with POP.

## 2 Materials and methods

A retrospective chart review study of women with POP was conducted at Kaohsiung Veteran General Hospital between January 2015 and June 2018. Institutional Review Board approval for the study was obtained (IRB No 18-CT6-17/180430-1). All women with stage II or more symptomatic POP who started to receive vaginal pessary treatment from January 2015 to December 2016 were enrolled. For those patients who had neurological diseases such as Parkinson disease or Alzheimer disease, or not able to take care of the pessary by themselves, were not eligible to pessary use. According to the chart review, there were no patients who had allergy to silicon. Women with the following conditions were excluded for further analysis

(1) failure of initial pessary fitting,
(2) incomplete initial data collection, such as initial pelvic organ prolapse quantification (POP-Q) record and evaluation of SUI,
(3) with a history of previous surgery for POP.

The basic patient characteristics including age, parity, body mass index, and the past history of medical diseases were collected at the time of pessary fitting.

Prolapse stage assessment was according to the POP-Q system.^[17]^ All enrolled women were required to do 1-hour pad test after reduction of POP by vaginal pessaries, and the method was according to the International Continence Society instructions.^[18]^ All nurses involved in performing the 1-hour pad test had received previous training from the primary investigator. One-hour pad test more than 10 gm were defined as severe SUI.^[19,20]^ Once these women underwent successful pessary fitting for 1 month, and were willing to continue vaginal pessaries, they would be followed up every 3 to 6 months regularly. We recorded their condition to June 2018 by chart review. The medical chart information included initial symptoms and subsequent change, pelvic examination results, any complications of pessary treatment, and whether they continued vaginal pessaries.

The outcome measures and associated clinical variables were analyzed using the chi-square test, Mann–Whitney statistical tests, 2-tailed Student *t* test, Kaplan–Meier survival analysis, and Cox regression analysis as appropriate. The Statistical Package for Social Sciences (version 20, Chicago, IL) was used for statistical analysis. A *P* value of .05 was considered to be significant.

## 3 Result

**Figure 1:**
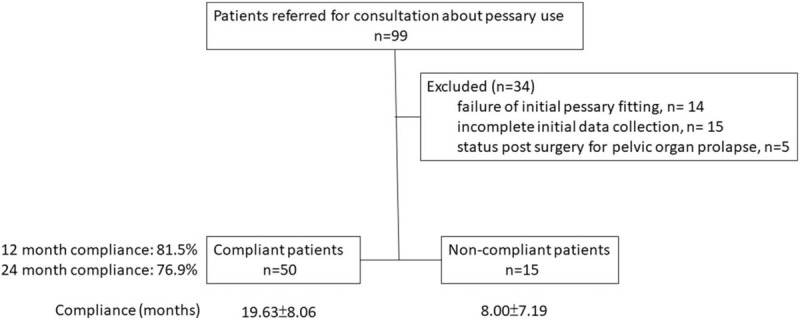
Study design.

Figure 1 was the flow diagram of patients through this study. From January 2015 to December 2016, a total of 99 women with symptomatic stage II or more POP underwent pessary fitting in our hospital. Thirty-four patients were excluded due to follow-up period less than 1 month (n = 14), incomplete basic data collection (n = 15), or history of previous surgery for POP (n = 5). The final analysis included 65 patients; 50 of them continued the vaginal pessary treatment (the compliant group) at the end of the study period, while 15 patients quit (the noncompliant group) (Fig. 1).

The baseline characteristics were similar between the 2 groups, except the age of women was younger in noncompliant group than compliant group (64.7 ± 10.9 vs 71.0 ± 8.2, *P* = .019^∗^) (Table 1). In our study, the overall 1-year compliance of vaginal pessary treatment was 81.5% and 2-year compliance was 78.5%. Women who suffered from severe SUI after reduction was prone to be noncompliant (40% vs 16%, *P* = .047).The majority of patients who stopped using pessaries underwent surgery instead (73.3%) (Table 2). There was no one who stopped pessary treatment due to complications.

**Table 1:**
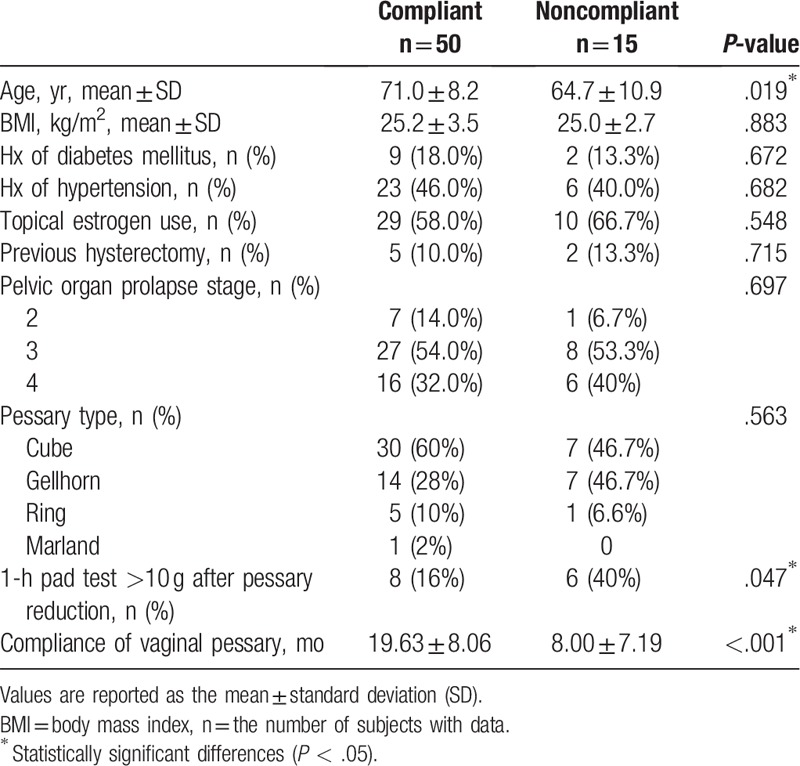
Patient demographics.

**Table 2:**
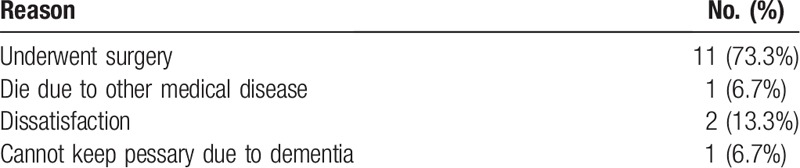
Outcomes of noncompliant patients.

**Figure 2:**
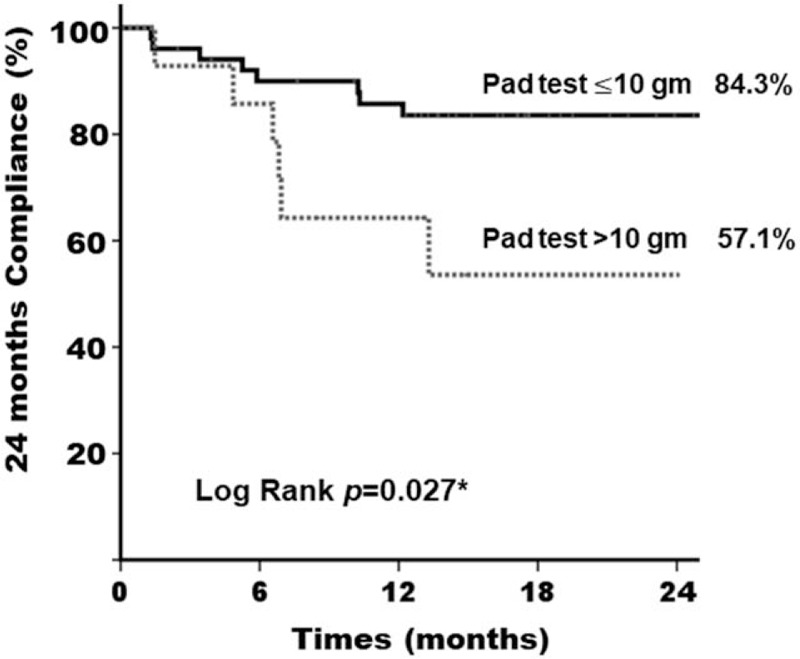
Kaplan–Meier curve at 24 mo after pessary treatment for women with pelvic organ prolapse. Comparison of women with stress urinary incontinence pad test >10 gm and pad test ≦10 gm.

**Figure 3:**
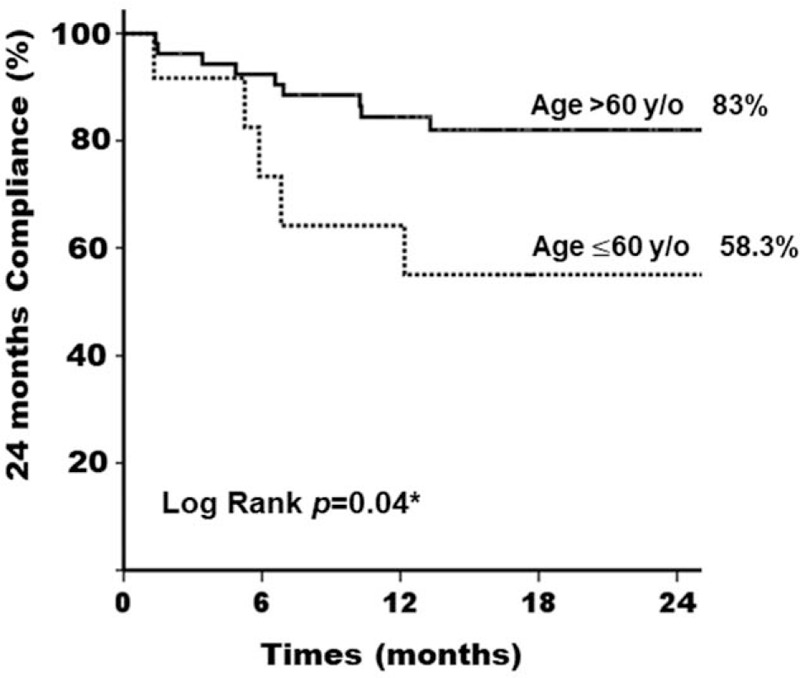
Kaplan–Meier curve at 24 mo after pessary treatment for women with pelvic organ prolapse. Comparison of women with age >60 yr old and ≦60 yr old.

**Figure 4:**
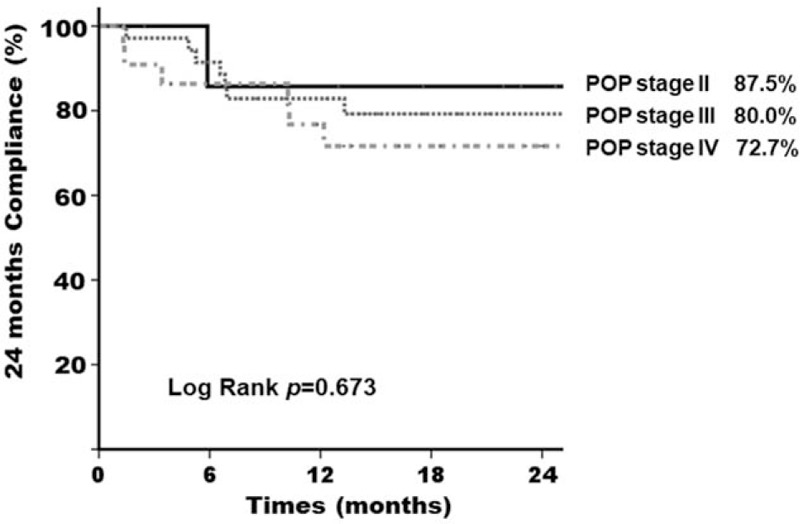
Kaplan–Meier curve at 24 mo after pessary treatment for women with pelvic organ prolapse. Comparison of women with stage II, stage III, and stage IV pelvic organ prolapse.

We found that 2-year compliance was poor in women with severe SUI after reduction and a Kaplan–Meier graph of the compliance revealed significant difference (pad test >10 gm vs pad test ≦10 gm, 57.1% vs 84.3%, *P* = .027^∗^) (Fig. 2). We further investigated other factors which may affect the long-term compliance of pessary treatment. Women who were younger than 60 years-old also showed poor pessary compliance compared to those older than 60-years-old (58.3% vs 83.0%, *P* = .04^∗^) (Fig. 3). However, POP stage did not show influence on the 2-year compliance in our study (*P* = .673) (Fig. 4). Therefore, we found that severe SUI after reduction (1-hour pad test >10 gm) and younger age (age ≦60-year-old) may result in a negative impact on the long-term compliance.

In order to analyze the factors which contribute to the noncompliance of pessary treatment, we performed multivariable cox regression analysis. We found that patients with severe SUI (1 hour pad test >10 gm) after pessary reduction predicted noncompliance with pessary treatment at 2-years as compared with patients of mild or no SUI symptoms (hazard ratio [HR], 7.11; 95% confidence interval [CI], 1.939–26.090; *P* = .003) (Table 3). Women with elderly age protected against noncompliance (HR, 0.915; 95% CI, 0.865–0.968; *P* = .002). No other variables, including POP stage, previous hysterectomy or pessary type, predicted noncompliance with follow-up.

**Table 3:**
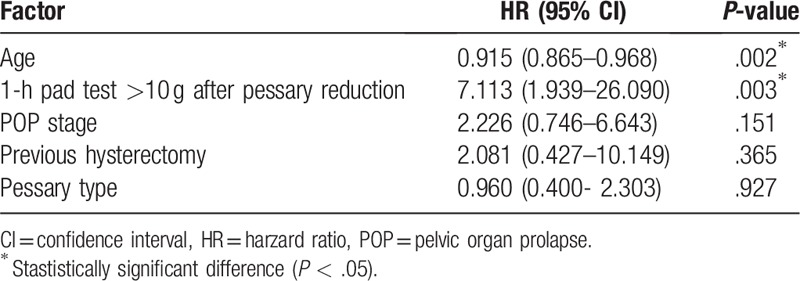
Analysis of factors that contribute to the noncompliance of pessary treatment.

## 4 Discussion

Vaginal pessary treatment for POP has been used for a long time either as an alternative to surgery or as a transient way to control symptoms. The common complications were extrusion of the pessary, bleeding, pain, or vaginal discharge, but these conditions could be easily solved after topical antibiotics, vaginal estrogen cream, or discontinuation of pessary for a few days.^[21]^ Since the pessary treatment is safe and effective, it is worth to find out the factors which may affect the long-term compliance.

Our study found that severe SUI after reduction of POP may decrease long-term compliance of vaginal pessaries at 24 months (*P* = .027). Elderly women who are more likely to have medical contraindications for surgery would keep good long-term compliance of vaginal pessaries (*P* = .04). Multivariate Cox regression analysis also revealed similar results. Therefore, we suggest that long-term pessary treatment would be more suitable for elderly patients and for those with no SUI or mild SUI symptoms. Evaluation of the severity of SUI after reduction before vaginal pessary treatment is warranted.

Urinary symptoms are often the most bothersome symptoms to women with POP. Usually, patients with obstructive symptoms may have significant improvement after pessary treatment, but to those women with severe SUI, the condition can get worse.^[2,14]^ Clemons et al had reported that dissatisfaction of pessary treatment was associated with occult SUI after a short-term pessary fitting for 2 months.^[9]^ Most women who were not satisfied and quitted the pessary treatment would undergo surgery instead. Hence, if we can have better understanding of the patients’ urinary problems, we would be able to provide proper consultation during the follow-up. One-hour pad test was still considered as a feasible test for evaluation of SUI in nowadays.^[20]^ Although only about half of the patients received conventional urodynamic study in our study, all patients had uroflowmetry, postvoiding residual urine, and 1-hour pad test, in prolapse and reduction status in order to evaluate urinary condition before and after pessary treatment. Compared to conventional urodynamic study which is invasive and expensive, and to 24-hour pad test which is time-consuming, 1-hour pad test appeared to be well-tolerated and accepted to every patient for the evaluation of SUI.

Our study showed that long-term vaginal pessary treatment is feasible and safe, especially acceptable for elderly patients. This finding is consistent with previous studies.^[3,7,22]^ They favored the pessary treatment may be because of their comorbidities or the absence of sexual activity.^[6,7,23]^ However, we still encourage younger patients who still have to do weight lifting jobs to keep pessary treatment. Persistent abdominal pressure due to weight bearing may lead to early recurrence of POP after surgery.

The stage of POP did not appear to be a determining factor for long-term compliance of vaginal pessaries in our study. Although patients with more severe POP may tend to require surgical intervention, we still can suggest every patient to start pessary treatment as the first-line therapy. As for the 4 different types of pessaries used in this study, we did not found correlation between the types of pessaries and long-term compliance. During initial pessary fitting, we selected the inserted pessaries for the patients by 3 experienced continence nurses according to the patients’ condition and preference. And then, each of the patients borrowed 1 pessary home, and was allowed to come back to change a different type or size of pessary if there was any problem. After 1 month of pessary fitting, the patient herself should decide 1 specific pessary and keep it ever after. It was also worth mentioned that although there were 4 different types of pessaries in this study, we did not use any anti-incontinence pessaries which might affect the severity of SUI after reduction. According to our multivariable cox regression analysis, and there was no correlation between the different types of pessaries and long-term compliance.

Previous hysterectomy was considered to be a less favorable condition in patients treated with pessaries.^[13]^ It was reported that vaginal estrogen cream could help women continue pessary use.^[24]^ In our study, we found both of these 2 factors did not affect our patients’ long-term compliance. It remained controversial whether to give the patients consistent use of vaginal estrogen cream or not. Some authors said that it is beneficial owing to the relief of atrophic vaginitis,^[24]^ while others showed no significant difference after 24 weeks of treatment.^[25]^

Our major achievements were comprehensive analyses of possibly related factors to the long-term compliance of vaginal pessaries, especially the success in demonstrating clinical correlation of severe SUI after reduction to noncompliance of pessary treatment. One-hour pad test is an easy and noninvasive way to objectively evaluate the patients’ severity of SUI after reduction. This gives us an objective evaluation method to predict the patients’ long-term compliance of pessary treatment. However, there were some limitations in this study. This was a retrospective study, and a big proportion (34/99, 34.3%) of the patients were excluded due to missing basic data, loss to follow up, or previous POP surgery. Additionally, 1-hour pad test was performed to every patient only during initial pessary fitting, and this may underestimate the patients suffered from severe SUI. The results would be more convincing if we completed urodynamic study and regularly followed up 1-hour pad test during every visit. However, because this was a retrospective study, only near half of the patients received complete urodynamic study, which is not sufficient for analysis. Meanwhile, we only repeated urodynamic study or pad test when it was required (eg, the patients complained about progressive symptoms of SUI, or asked for surgical intervention). Thus, we could not present the change of SUI after long-term pessary treatment. There is still 1 thing to be stressed, although we consider severe SUI after reduction and younger age are factors of poor long-term compliance of vaginal pessaries, pessary treatment should not be discouraged to these women since the satisfaction is case by case.

In conclusion, severe SUI after reduction (1-hour pad test >10 gm) or younger age (age ≦60-year-old) were significantly associated with poor long-term compliance in women with POP treated with vaginal pessaries. Objective evaluation of SUI is important to predict patients’ compliance and help us to provide more information during consultation. Additional large, well-designed prospective studies are worthwhile and necessary.

## Author contributions

MF Hsieh, CC Lo, YF An, and ZH Lin were responsible for extracting the data. MF Hsieh, HW Tsai, and HY Lin were responsible for the analysis and interpretation of data. HW Tsai and HY Lin were drafting the article. WS Liou and HY Lin were responsible for the design of the study and the acquisition of data. HW Tsai and HY Lin were responsible for revising the manuscript and for the final approval of the version to be submitted.

**Conceptualization:** Hsiao-Wen Tsai, Hsin-Yin Lin.

**Data curation:** Ming-Fang Hsieh, Ching-Chuan Lo, Zi-Han Lin, Ya-Fen An.

**Formal analysis:** Ming-Fang Hsieh, Hsin-Yin Lin.

**Investigation:** Wen-Shiung Liou, Ching-Chuan Lo, Zi-Han Lin.

**Methodology:** Ming-Fang Hsieh, Ya-Fen An.

**Supervision:** Wen-Shiung Liou.

**Writing – original draft:** Hsiao-Wen Tsai.

**Writing – review and editing:** Hsiao-Wen Tsai, Hsin-Yin Lin.

Hsiao-Wen Tsai orcid: 0000-0003-3483-3943.
